# Preliminary Observations on *Zelus obscuridorsis* (Stål) (Hemiptera: Reduviidae) as Predator of the Corn Leafhopper (Hemiptera: Cicadellidae) in Argentina

**DOI:** 10.3390/insects6020508

**Published:** 2015-06-03

**Authors:** Eduardo G. Virla, Cecilia M. Melo, Stefano Speranza

**Affiliations:** 1 CONICET, Fundación M. Lillo (Instituto de Entomología), San Miguel de Tucumán T4000JFE, Argentina; E-Mail: evirla@hotmail.com; 2 CONICET, División Entomología, Museo de Ciencias Naturales de La Plata, La Plata B1900FWA, Argentina; E-Mail: melo.cecilia@gmail.com; 3 Department of Agriculture, Forests, Nature and Energy, University of Tuscia, Viterbo 01100, Italy

**Keywords:** assassin bug, *Dalbulus maidis*, biological control, corn diseases, vector

## Abstract

The corn leafhopper *Dalbulus maidis* (Hemiptera: Cicadellidae), is an important corn pest in most of tropical and subtropical America. This leafhopper has a rich natural enemy complex of which parasitoids and pathogens are the most studied; knowledge on its predators is limited. We noted the presence of the native assassin bug *Zelus obscuridorsis* (Hemiptera: Reduviidae) predating diverse motile insects, including the corn leafhopper, on corn plants cultivated in household vegetable gardens in San Miguel de Tucumán (Argentina); in order to verify its predatory actions, we exposed lab-bred individuals of *D. maidis* to adults of *Z. obscuridorsis*. The predators were starved for 24 h before trials in which the corn leafhopper in different developmental stages were exposed. *Zelus obscuridorsis* is highly skilled in catching specimens in motion, but it was not able to prey on eggs. The predator was capable to catch and prey on nymphs and adults.

## 1. Introduction

The corn leafhopper *Dalbulus maidis* (DeLong and Wolcott) (Hemiptera: Cicadellidae), causes serious damage to corn crops in most of tropical and subtropical America. It only feeds on plants of the genus *Zea* (maize and teosintes) [1]; and it efficiently transmits three important plant pathogens: Corn stunt spiroplasma (CSS), maize bushy stunt phytoplasma (MBSP) and maize rayado fino virus (MRFV) [2]. The distribution of the CSS has increased significantly in South America [3]. In Argentina, CSS disease is present with incidences of up to 100% in the subtropical region [4,5].

The corn leafhopper has a rich natural enemy complex, the most studied of which are parasitoids and pathogens [6]. Regarding predators, knowledge is scarce and fragmentary. In Argentina, only a species of earwig [*Doru lineare* (Eschscholtz), Dermaptera: Forficulidae] have been reported affecting the eggs of *D. maidis* [7]. In Ecuador, a technical report recorded spiders (Salticidae), assassin bugs (*Zelus* sp., Hemiptera: Reduviidae), ladybird beetles [*Hippodamia convergens* (Guérin-Méneville) and *Cycloneda sanguinea* (Linnaeus), Coleoptera: Coccinellidae], lacewings (Neuroptera), and wasps (*Polybia* sp. and *Polistes* sp., Hymenoptera: Vespidae) [8]. In Mexico, other species of *Dalbulus* (the myrmecophile *D. quinquenotatus* DeLong and Nault) is attacked by spiders (Anyphaenidae, Philodromidae, Thomisidae and Araneidae) and by the generalist nabid predator *Nabis americoferus* Carayon [9,10]. It should be noted, moreover, that the Dryinidae (Hymenoptera: Chrysidoidea) are parasitoids of leafhoppers and the females of *Gonatopus bartletti* Olmi, on *D. maidis* can actively conduct host-feeding behaviours also [11].

Assassin bugs are polyphagous [12], and some species have proven to be efficient predators on insect pests of several crops, playing a significant role in decreasing pest populations [13]. While monitoring infested corn plants cultivated in household vegetable gardens at San Miguel de Tucumán (Argentina), during the middle of spring 2012 (Oct), we noted the presence of a native assassin bug predating diverse mobile insects like aphids, flies and corn leafhoppers. We identified this predator as *Zelus obscuridorsis* (Stål) (Hemiptera: Reduviidae), a poorly known species already recorded from Argentina [14].

Considering the relevance of the corn leafhopper and the economic importance of the transmitted diseases, there is an increasing interest to develop biological control and/or integrated management programs [15]. Moreover, the need for knowledge of interrelationships of insect pest and its native antagonists in order to develop effective management tactics has been highlighted by several authors. In this context, the aim of this contribution is to assess the predatory potential of *Z. obscuridorsis* on different stages of the corn leafhopper.

## 2. Materials and Methods

In order to test the ability of the predator to catch and prey on *D. maidis*, we exposed lab-bred individuals of *D. maidis* at different stages of development to *Z. obscuridorsis* adults.

Specimens of the predator were collected in the field (San Miguel de Tucumán, Tucumán Province, Argentina S 26°48'35.6'', W 65°14'24.6'', 500 m asl), and were brought to the laboratory and maintained individually in breeding cages (30 × 30 × 20 cm), made of aluminum with lateral and the upper side covered with organdy type mesh to promote ventilation, with a fresh corn leaf inside.

A *D. maidis* colony was established with individuals collected during the summer of 2013 in Los Nogales, Tucumán Province, Argentina (26º42' S, 65°13' W; 588 m asl). The adults were placed in breeding cages (50 × 50 × 50 cm), made of aluminum with the lateral and upper sides covered with nylon mesh (organdy type) to promote ventilation. Potted corn plants (pot of 6.3 dm^3^) were placed inside as food source and for reproduction. The maize variety “Leales 25 plus” was used both for maintenance of the corn leafhopper colony and for all the assays. The colony was reared in a greenhouse at San Miguel de Tucumán (S 26°4835.6', W 65°1424.6'; 500 m asl) under the following conditions: temperature between 20 and 30 °C, the natural photoperiod, and no humidity control.

The predators were starved for 24 h before trials and were randomly collected from the breeding cages. All tests were conducted in laboratory at 25 ± 3 °C, 70% ± 20% RH and the natural photoperiod (near 13:11 h L:D). The size of the arena for determining parasitism or prey consumption rates is important [15]; for reduviid predators, some authors used Petri dishes (200 mm diameter) [16], but as we needed to introduce a leaf of corn as food for the leafhoppers we used cubic glass arenas (500 cm^3^, 20 × 5 × 5 cm). The glass cages were opened at both ends, one of them covered with a nylon mesh for aeration, and the other capped with a cubic piece of polyurethane foam, through which a fresh maize leaf was introduced.

The number of assays with different stages of *D. maidis* is summarized as follows: (a) four replicates of leaves containing 19, 24, 24, and 30 eggs respectively; (b) six replicates of six small nymphs (I and II instars); (c) six replicates of six large nymphs (IV or V instars); (d) six replicates of six adults (three males and three females). In each replicate, one adult was allowed to prey in the arena for 6 h. After assays were completed, living preys were counted.

Because normality and homogeneity of variance tests (Shapiro-Wilks) did not pass, the number of small, and large nymphs, and adults of *D. maidis* preyed in the assays were compared with Kruskal Wallis test. When comparing the number of adults attacked by sex, the normality test passed and therefore the means were compared using a *t* test. Statistical analyses were performed using INFOSTAT version 2013 [17].

Voucher specimens of *D. maidis* are deposited in the M. Lillo Foundation (IFML) and of *Z. obscuridorsis* at the Museo de La Plata (MLP).

## 3. Results

No eggs of the corn leafhopper in the four trials were attacked. The predator was only capable to catch and prey on nymphs and adults, but its ability to capture them differed. *Zelus obscuridorsis* preyed on 8.3% of the small nymphs offered, 19.4% of the large nymphs, and 52.7% of the adults. The mean number of preyed life stages were 0.5 small nymphs/ trial (SE: 0.28; range: 0–1), 1.17 large nymphs/trial (SE: 0.28; range: 0–2) and 3.17 adults/trial (SE: 0.29; range: 2–4). Adults were more frequently captured than both classes of nymphs, but there was no statistically significant difference between large and small nymphs (Kruskal-Wallis test, H: 11.8, df = 2, *p* < 0.05).

The predator did not show any tendency to differentially attack either sex of the corn leafhopper (*t* test, df = 10, *p* = 0.69), attacking 47.4% and 52.6% of males and females, respectively (nine males from 18 and 10 females from the 18 exposed individuals). All dead preys showed clear signs of predatory actions (e.g., wounds in their abdomens) and none died from other causes (e.g., starvation, desiccation).

**Figure 1:**
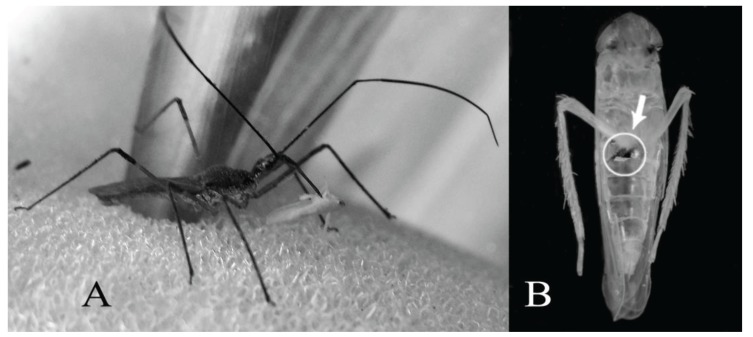
(**A**) *Zelus obscuridorsis* (Stål) preying an adult of *Dalbulus maidis* (DeLong and Wolcott); (**B**) corn leafhopper’s empty exoskeleton; the arrow indicates the area of insertion of the stylets.

By direct observation in the field, we have detected on two occasions individuals of the reduviid preying adults of the corn leafhopper. This allowed us to notice that the adults of *Z. obscuridorsis* are highly skilled in catching specimens in motion. The act of predation involves the adoption of specific body postures of the reduviid. While waiting for a prey, it stands on its median and hind legs keeping the forelegs forward and up with the antennae in the same position; when a leafhopper is detected, it catches the prey with its forelegs and pierces it with the stylets (Figure 1A). Once the stylets are introduced, *Z. obscuridorsis* feeds on a leafhopper for 5–10 min and then discards the leafhopper’s empty exoskeleton (Figure 1B).

As mentioned above, the lack of knowledge about the species that act as *D. maidis* predators is significant. This is emphasized in Argentina where only an earwig is known to prey on the eggs of *D. maidis* while no species, until now, has been reported attacking their nymphs and adults. Our results allow us to state that *Z. obscuridorsis* is a generalist predator that is proficient in capturing *D. maidis* adults, is less efficient capturing the nymphs, and it is not able to prey on the vector's endophytic eggs. The nymphs of *D. maidis*, mostly the smaller ones, are generally very still feeding on the midrib of the leaves, this showed us that *Z. obscuridorsis* is very skilled in trapping preys in motion. It should be noted that motile preys can be hard to manipulate by relatively small-bodied predators (such as mirids, anthocorids or coccinelids) [18,19], therefore, the performance of this large predatory bug (*ca*. 11 mm) should be taken into consideration. Life stages studies [20,21] revealed that each stage of a reduviid prefers a particular stage of the prey: large sized predators prefer larger sized prey and smaller sized predators prefer smaller sized prey [13].

## 4. Conclusions

This study confirms that *Z. obscuridorsis* (Hemiptera: Reduviidae) is an active predator of the corn leafhopper that is highly successful at capturing motile stages. The obtained results are a starting point to evaluate its abundance, biology and behaviour in corn crops; and further research is needed to increase the understanding of the role of this generalist predator in *D. maidis* natural control.
